# T Lymphocyte Exhaustion During Human and Experimental Visceral Leishmaniasis

**DOI:** 10.3389/fimmu.2022.835711

**Published:** 2022-05-02

**Authors:** Juliana C. Costa-Madeira, Gabrielly B. Trindade, Paulo H. P. Almeida, João S. Silva, Vanessa Carregaro

**Affiliations:** ^1^Department of Biochemistry and Immunology, Ribeirão Preto Medical School, University from São Paulo, Ribeirão Preto, Brazil; ^2^Fiocruz-Bi-Institutional Translational Medicine Project, Ribeirão Preto, Brazil

**Keywords:** visceral leishmanaisis, T cell exhaustion, Th subsets, inflammation, inhibitory receptor

## Abstract

A key point of immunity against protozoan *Leishmania* parasites is the development of an optimal T cell response, which includes a low apoptotic rate, high proliferative activity and polyfunctionality. During acute infection, antigen-specific T cells recognize the pathogen resulting in pathogen control but not elimination, promoting the development and the maintenance of a population of circulating effector cells that mount rapid response quickly after re-exposure to the parasite. However, in the case of visceral disease, the functionality of specific T cells is lost during chronic infection, resulting in inferior effector functions, poor response to specific restimulation, and suboptimal homeostatic proliferation, a term referred to as T cell exhaustion. Multiple factors, including parasite load, infection duration and host immunity, affect T lymphocyte exhaustion. These factors contribute to antigen persistence by promoting inhibitory receptor expression and sustained production of soluble mediators, influencing suppressive cell function and the release of endogenous molecules into chronically inflamed tissue. Together, these signals encourage several changes, reprogramming cells into a quiescent state, which reflects disease progression to more severe forms, and development of acquired resistance to conventional drugs to treat the disease. These points are discussed in this review.

## Introduction

Leishmaniasis is a set of anthropozoonotic diseases caused by several species of trypanosomatids of the *Leishmania* genus, comprising species responsible for different pathologies (1). Clinically, leishmaniasis can manifest in tegumentary (localized, disseminated or diffuse), mucocutaneous and visceral forms. The taxonomy of *Leishmania* correlates a particular species with a particular clinical manifestation in humans. The parasite species of the *Leishmania mexicana* and *Leishmania braziliensis* complex (in the New World) and *Leishmania major* and *Leishmania tropica* (in the Old World) are related to cutaneous and mucocutaneous forms, while the parasites of the *Leishmania donovani* complex, the species *Leishmania donovani* and *Leishmania infantum*, are associated with the visceral form of the disease. Visceral leishmaniasis (VL), or kala azar, is the most severe form of disease. The disease can be caused by *L. donovani* in India and sub-Saharan Africa and *L. infantum* in southern Europe, North Africa and Brazil (2, 3). The disease is characterized by spreading of parasites to the viscera (mainly spleen, liver and bone marrow). The clinical forms are diverse, and affected individuals may present symptoms ranging from spontaneous cure and oligo and asymptomatic forms to severe manifestations, which may lead to death (4). The symptomatological spectrum of the disease is characterized by dissemination of parasites to the organs (5). Symptoms such as prolonged fever, asthenia, anorexia, weight loss, hepatosplenomegaly, pancytopenia, hypergammaglobulinemia and severe anemia are present in individuals with the disease. In the most severe form of VL, alopecia and edema of the lower limbs can be observed, as well as hemorrhagic manifestations, such as epistaxis, ecchymosis and petechiae, as a result of liver alterations and thrombocytopenia resulting from pathological liver changes (6). Another important aspect is the development of neutropenia, which promotes host susceptibility to bacterial infections (7). Together, these signs and symptoms may intensify the severity of disease and lead to patient death if not properly treated. Current treatment options are far from ideal, with diverse outcomes based on multiple factors, including geographic location, immune status, disease stage, and patient comorbidities. Current first-line treatments for VL, such as amphotericin B (liposomal or deoxycholate formulations), miltefosine, paromomycin, and antimonials, are far from ideal for use in resource-poor settings due to issues such as teratogenicity, clinical resistance and/or relapse, the long-term nature of the treatment regimens and parenteral administration (4, 8). These findings suggest that exploring the therapeutic potential of immunomodulatory drugs for the treatment of VL, mainly in areas of anthroponotic transmission (transmitted by only humans), is warranted (9).

## Immune Response During VL

It has been well established in a murine model that host resistance is related to CD4^+^T cells producing IFN-γ (T helper 1 (Th1) subset). Dendritic cells (DCs) capture parasites at the site of infection and migrate to draining lymphoid organs, where they stimulate the differentiation of naïve CD4^+^T cells to differentiate into the Th1 subset in a manner dependent on IL-12 production (10). Along with CD4^+^T lymphocytes, natural killer (NK) cells and CD8^+^T cells are also important sources of IFN-γ (11). Such cytokines act on macrophages, activating inducible nitric oxide synthase (iNOS) with concomitant production of nitric oxide (NO), leading to the death of phagocytosed parasites (12). TNF is produced by infected macrophages and acts with IFN-γ, increasing iNOS activation and thus leading to NO-mediated parasite death (13–15). Furthermore, Th17 response controls infection by acting in synergism with IFN-γ, increasing NO production by macrophages, and promoting neutrophil influx into the target organs of the disease (16, 17). Conversely, the Th2-type response is related to susceptibility and disease progression (18–20). A microenvironment with a predominance of IL-4, IL-13 and IL-10 induces the differentiation of naïve CD4^+^ T cells into the Th2 subtype, promoting parasite survival (21). Leishmania parasites can use monocytes to induce the infection establishment into the host, as mechanisms of subversion of the immune response and promoting parasites survival. In this sense, it has been demonstrated that *L. donovani* induces the expansion of hematopoietic stem cells (HSCs) and differentiation of GMP (granulocyte-monocyte progenitors) cells into Ly6C^hi^ (an inflammatory monocytes) with a more permissive profile to infection, characterized by the expression of regulatory markers such as Sca1, Galactin-3, MHC-II and IL-10 (22). In addition to Kupffer cells and macrophages from the marginal zone of the spleen, *L. donovani* also infects these Ly6C^hi^ recruited to the spleen and liver during infection and compromises the ability of these cells to control parasites, which contributes to susceptibility to disease (23, 24).

As mentioned above, hepatosplenomegaly is a hallmark of VL. This symptom results from an imbalance between the immune response that controls parasite replication and those that allow the pathogen to persist. In experimental models, the infection resolves within 3 months. This resolution is related to the development of well-formed granulomas by Th1 cells (25). Neutrophils and macrophages surround infected cells (macrophages and Kupfer cells) and are activated by IFN-γ, leading to NO release from phagocytes and promoting parasite restriction (26, 27).

Both Th1 and Th17 immune responses are driven by innate molecules, receptors such as TLRs (TLR2, TLR7, TLR8, and TLR9) and NLRs (NOD2 and NLRP3), and cytokines (IL-1β, IL-6, IL-12, IL-23 and IL-18) expressed by DCs, which influence parasite restriction (28–30). Other innate receptors, such as TLR4, are exploited by parasites to both subvert the Th1 immune response and successfully establish themselves in the vertebrate host (31). Moreover, the signaling triggered through NOD2-RIP2 induces a Th1 response but inhibits genes related to the Th17 subtype in a murine model and in leukocytes recovered from VL patients (32). An interesting issue is that, in general, VL-causing species are less inflammatory than cutaneous leishmaniasis-causing species (33). Thus, in the early stages of the disease, the liver is less affected and suffers damage with chronicity. This promotes disease progression without promoting parasite elimination.

## Chronicity During VL: Exhaustion of T Lymphocytes

**Figure 1:**
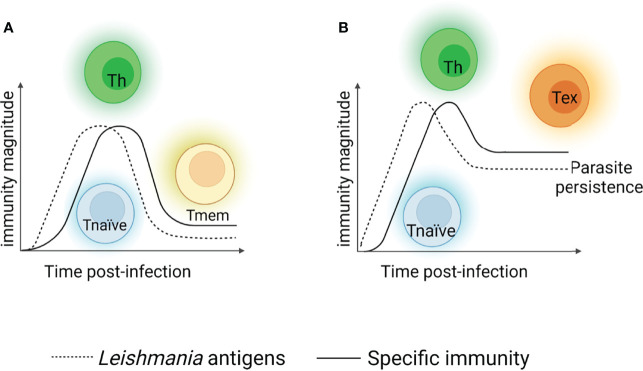
T cell response after viscerotropic leishmania parasites recognition. When *L. donovani* or *L. infantum* antigens are recognized, specific naïve CD4^+^ T cells (blue cells) are activated and differentiate into distinct subtypes of T helper lymphocytes (Th1, Th17 – green cells), restricting the parasites replication. After parasite declining, the most of Th cells die and the remaining cells differentiate into memory T cells (yellow cells). In the case of secondary exposure to the microorganism, memory T cells may be reactivated and promoting parasite control **(A)**. Situations where the specific immune response generated in the host cannot control the parasite, a chronic infection process is established. The parasite’s persistence induces Thelper cells to enter a non-functional state, a term referred as exhaustion (represented by orange cells), rather than developing a classical memory cells. Exhausted T cells present a compromised effector functions and maintain the persistence of the parasites in the host for long periods, which reflects disease progression to more severe forms, but it was not found it in asymptomatic individual or patients with mild form of disease **(B)**. Created with BioRender.com (Agreement number: IK23QKCBAC).

During the acute phase of *Leishmania* infection, specific T cell populations undergo a dramatic numerical increase and differentiate to cells with appropriate effector functions for declining parasite number. This process is usually followed by a substantial loss of effector cells but maintains an elevated number of memory T cells, which can be efficiently deployed when an individual is reinfected by the parasite (**Figure 1A**). However, several patients become immunosuppressed and may succumb to secondary infections. This inability to control visceral infection has been attributed to a defect in generating a cellular immune response, which enables parasite survival (34, 35). Persistent exposure to antigens and/or chronic exuberant inflammation alters several T cell functions, mainly those of memory cells, and is one of the causes of immunopathology development that occurs during the active form of the disease. This process is known as cell exhaustion, and it precludes an effector immune response for long periods. The cell exhaustion process is followed by an increase in parasite load. In such scenarios, the impairment of T cell effector functions is supported by the increase and coexpression of several inhibitory receptors, cytokines and endogenous molecules that promote several alterations, reprogramming cells to a quiescent state (**Figure 1B**).

Although the exhaustion term was first described during viral infections, we recognize that parasites evoke a robust immune response in the host that often fails to eliminate the pathogen (33). In this sense, the participation of exhausted T cells in the immunopathogenesis of leishmaniasis has been investigated. The presence of T cells with exhaustion phenotypes has been described in infections caused by species that cause visceral disease and those that cause the cutaneous manifestations. Interestingly, the exhausted T cells seem to affect the outcome of the disease differently depending on the species of *Leishmania* involved. During *L. mexicana* infection, dendritic cells produce high amounts of TNF, contributing to the T cells exhaustion, compromising the proliferation and functionality of these cells, which favors the progression of the disease (36). Patients infected by *L. braziliensis* show increased expression of exhaustion markers on CD4^+^ T cells and CD8^+^ T cells in the skin and in the bloodstream as well. However, the extent of the lesion is not related to the expression of inhibitory molecules such as PD-1, suggesting that exhausted T cells do not interfere in the pathogenesis of the disease (37). In *L. major* infection, characterized by lesions that heal spontaneously in most affected individuals, the infection is controlled within 3-8 weeks. However, no sterile cure is observed and the persistence of remaining parasites, controlled in part by CD4^+^CD25^+^ regulatory T cells, appears to be crucial for the maintenance of short-lived CD4^+^ effector T cells, protecting the individual from reinfection. This protection is lost when the parasites are controlled, suggesting that antigenic persistence is necessary for long-lasting immunity, although it can induce exhaustion phenotype in antigen-specific cells (38–40). Conversely, the persistence of the parasites in visceral leishmaniasis promotes a dysfunctional response of CD8^+^T lymphocytes, which encourages parasite survival and replication. Even though CD8^+^T cells are increased in the blood and lesions of chronically infected patients, individuals with severe VL show impaired cellular proliferation and cytokine production (i.e., IL-2 and IFN-γ by these cells. In a VL experimental model induced by *L. donovani*, CD8^+^T lymphocytes do not undergo significant proliferation or activation, maintaining antigen persistence (41, 42). The presence of the parasite for a long period promotes the exhaustion of CD8^+^T lymphocytes, generating cells with limited capacity to produce IFN-γ, which leads to cell death (43). CD8^+^T cells from transgenic *L. donovani* expressing ovoalbumin cells exhibited a biphasic wave of activation, with the first wave being involved in a limited expansion and the second wave resulting in the death and exhaustion of CD8^+^T lymphocytes. Peripheral blood-derived mononuclear cells (PBMCs) recovered from *L. infantum*-infected patients with the severe disease neither proliferate nor produce IFN-γ after *in vitro* soluble *Leishmania* antigen (SLA) stimulation. PBMCs isolated from healed VL patients respond to specific antigens and produce IFN-γ, suggesting that reversal of the profile of exhausted cells can lead to a favorable clinical outcome (44–46). It is important to consider that the field of CD4^+^T lymphocyte exhaustion is less explored, as from an evolutionary point of view, CD8^+^T lymphocytes are more vulnerable than CD4^+^T cells to tolerogenic stimuli, including prolonged persistence of specific antigen stimulation during chronic infection. This scenario protects the infected cells from cytotoxic responses and significantly reduces the inflammatory stimuli that could cause immunopathology in the host (47), and these mechanisms are promising topics to explore to increase the understanding of the physiopathogenesis of VL. Interestingly, different from cutaneous disease, where C57BL/6 or BALB/c strain may be employ as resistance and susceptible model of infection, respectively, the murine VL model is a clear example of organ-restricted immunity, where the parasites are naturally declined in long-term, without the development of clinical manifestations in mice strain-independent (26, 48). Although the most susceptible mouse strain, including BALB/c, developed a specific immunity, they may control parasites growth at longer term of infection. Thus, it may be plausible to consider the process of exhausted T cells could occur in mice strain independently in murine model of disease.

We do not discard the possibility that the failure to detect cellular-mediated immunity (CMI) in some sample of patients with the classical VL disease could be also an interference of the assay systems employed versus a lack of a CMI response. Although it has been demonstrated that the development of a Th1 response is necessary for the control of the parasite, recently it was described that patients with active VL present high levels of IFN-γ in the bloodstream, bone marrow, in addition to increased expression of such cytokine in organs such as the spleen and liver, suggesting that there is no defect in cell-mediated immunity in these patients (41, 49, 50). Furthermore, studies using whole blood assays show that, unlike from PBMC assays, CD4^+^ T cells from patients with active VL produce IFN-γ when re-stimulated *in vitro* with parasite antigens, in example, the absence of a response cell-mediated immune response observed in patients with active VL appears to be more due to the detection method employed than an actual failure of cellular immunity in VL generated (51, 52). In this sense, in humans, the disease progression appears to be unrelated to the production of IFN-γ and other mechanisms may be involved in the immunopathogenesis of the VL disease, such as cytokines, inhibitory molecules, endogenous mediators and suppressive cells. All these issues will be covered in this Review.

## Suppressive Cells

Given their ability to produce anti-inflammatory cytokines, to express high levels of inhibitory receptors, Tregs block the activation and function of innate and adaptive immune cells, promoting immune response control and maintenance of self-tolerance (53, 54). Despite the beneficial roles of Tregs in the host, including preventing the development of immunopathologies, their suppressive function can be exploited by some types of microorganisms to promote escape of the host immune response (55). This scenario is commonly seen during infections caused by *Leishmania* parasites (56).

The presence of both IL-10 and TGF-β cytokines in the infected tissue promotes the suppression of the protective immune response (i.e., Th1 cells), causing persistence of the parasite and chronicity of the disease, strongly suggesting the involvement of Tregs in the disease establishment (57–59). CD4^+^T cells often coproduce IL-10 and IFN-γ, designated type 1 regulatory T cells (Tr1s), and promote the onset of infection by suppressing Th1 cell-mediated immunity (50, 59, 60). The role of IL-10 and TGF-β in immunosuppression and disease progression is well documented in both experimental and human VL (9, 61). Regarding *L. donovani* infection, studies report that the increase in parasite number is related to the presence of Tregs and heightened levels of systemic IL-10 and TGF-β in patients with active disease. Corroborating the role of Tregs in disease progression, a reduced hepatic parasite load was observed in animals depleted of Tregs using both anti-CD25 and anti-FR4 neutralizing antibodies (62, 63). It was identified that such cells produce only TGF-β in a sustained manner, while the production of IL-10 was attributed to CD4^+^CD25^-^ T cells and DCs. TGF-β produced by Tregs was also observed in an experimental model of VL caused by *L. infantum.*

Despite not directly influencing parasite replication, Tregs play other roles, mainly in tissue protection and controlling leukocyte activation in both the initial and chronic phases of infection. These roles are independent of IL-10. In contrast, infection caused by *L. infantum* and TGF-β production by Tregs are related to the growth and persistence of the parasite, in addition to acting in the control of immunopathologies developed during infection (50, 59, 60, 62, 64). In experimental models of VL with *L. infantum* or *L. donovani*, treatment with CXCL10 promotes the protection of infected mice by stimulating the Th1 response, which decreases the population of Tregs and CD4^+^CD25^-^ T cells producing TGF-β and IL-10, leading to a reduction in the number of parasites in the spleen of treated animals and a consequent reduction in organ size (65, 66). Thus, Tregs contribute to T cell exhaustion by suppressing the effector functions of T lymphocytes and contributing to the persistence of the parasite.

## Inhibitory Receptors

Inhibitory receptors are negative regulators that control autoreactivity and prevent the development of immunopathology. Although some of these receptors are transiently expressed on functional effector T cells during activation, the high and sustained expression of inhibitory receptors is a hallmark of exhausted T cells. In experimental VL, it has been shown that chronic infection promotes the upregulation of several inhibitory receptor genes and some of their ligands. Programmed cell death protein 1 (PD1)-mediated inhibitory signaling in response to PDL1 and PDL2 provides a classic example by which this pathway manages T lymphocyte exhaustion. PD-1 is an inhibitory receptor and a member of the B-7 costimulatory receptor family expressed by all activated T cells, although it can also be expressed by other cell types (67). PD-1 regulates lymphocyte activation by binding to PD-L1 (B7-H1) and PD-L2 (B7-H2) ligands on lymphocytes and is essential for the maintenance of tolerance and homeostasis and the prevention (67, 68). Although the PD-1/PD-L1 or PD-L2 pathway plays an role in regulating the immune response magnitude, it may limit protective immunity against persistent antigens, a response observed in both cancer and chronic infection (69). It has been shown that experimental VL induced by *L. donovani* results in increased PD-1 expression by CD8*^+^* T cells with a phenotype of cell exhaustion, characterized by low expression of IL-12, IFN-γ and TNF, and blockade of PD-L1 partially recovers cell function (70). Spleen cell cultures from hamsters infected with *L. donovani* do not respond to parasite antigens. This unresponsiveness of CD4^+^T lymphocytes correlates with an increased production of regulatory cytokines, such as IL-10, TGF-β, IL-27, IFN-I, and inhibitory receptors, such as PD1 (71). *In vivo* blockade of PD1 using specific antibodies decreases arginase-1 expression in macrophages, resulting in a reduction in the parasite load of the organ (72). In addition to restoring the function of both CD4^+^ T and CD8^+^ T lymphocytes and decreasing parasite numbers, blocking the PD1/PDL1 pathway with anti-PDL1 antibodies reverses inhibition of caused by *L. donovani*, a mechanism used by the parasite to subvert the host’s autophagic machinery to encourage survival and induce the establishment of infection (73). In canine VL caused by *L. infantum*, PD1 blockade restores the effector functions of both CD4^+^ T and CD8^+^ T lymphocytes and the production of reactive oxygen species (ROS) by monocytes recovered from dogs with active VL, thus controlling the parasites replication (72). The interaction between PD1 and PDL1 increases the expression of FOXP3 and enhances the immunosuppressive activity of Tregs (74, 75). Furthermore, PDL1 converts naïve CD4^+^T cells into Tregs by downregulating AKT, mTOR and ERK2 and simultaneously upregulating PTEN (74). Thus, blocking the PD-1/PD-L1 axis reverses cellular exhaustion, exerts effects on peripheral tolerance by interfering with Treg induction and function, and may be a potential strategy for the treatment of patients with VL, mainly those unresponsive to conventional treatments.

There is currently a debate regarding the effect of immunotherapy that blocks the PD1/PDL1 or PDL2 axis in clinical oncology. Although highly effective for different types of cancer, rapid tumor progression was observed in approximately 10% of patients with advanced gastric cancer using an anti-PD1 monoclonal antibody (76–78). These patients had FOXP3^+^ Tregs with a high proliferation rate and increased suppressive function. Likewise, PD1-deficient murine Tregs are more proliferative and suppressive than wild-type Tregs isolated from the tumor (79). Evidence on the PD1/PDL1 interaction during Treg induction and function in VL is still scarce, but it is a topic that warrants investigation considering that blocking this pathway may generate a subversive response, maintain the persistence of the parasite, or increase the effector response and promote immunopathologies.

Cytotoxic T lymphocyte antigen 4 (CTLA-4) is another inhibitory receptor related to T lymphocyte expansion. It is a transmembrane glycoprotein homologous to the CD28 costimulatory receptor and essential for the regulation of the immune system. Both CD28 and CTLA-4 receptors share the same ligands, B7-1 (CD80) and B7-2 (CD86), expressed by antigen-presenting cells (APCs) during antigen presentation to T cells (80, 81). Competition between receptors for ligands reduces the number of specific T cells, which constitutes an important strategy for controlling the magnitude of responses in peripheral tissues to prevent the tissue damage (80, 82). The increased expression of such molecules has been demonstrated during acute infections and chronic infections, in which the antigen persists. Although this regulatory mechanism minimizes tissue damage, it can also compromise the elimination of pathogens, favoring their persistence in the host (83). During chronic VL infections, CTLA-4 is one of the markers of T cell exhaustion (84), and its role in susceptibility to infection in murine models, humans and dogs has been reported. CTLA-4 blockade during *L. donovani* infection increases parasite resistance in BALB/c mice, characterized by increased IFN-γ- and IL-4-producing cells and the rapid development of hepatic granulomas, which contain the spread of the parasite (85). Mice infected with *L. donovani* anti-CTLA-4 antibodies showed induction of increased leishmanicidal activity, IFN-γ production and increased hepatic granulomas compared to the untreated group (86, 87). Likewise, CD4^+^T cells recovered from the spleen of *L. infantum*-infected BALB/c mice show a poor proliferative rate in response to anti-CD3 stimuli and do not respond to *Leishmania* Lcr1 antigen, but *in vitro*, the blockade of CTLA-4 partially recovers the response (88). One suppressive mechanism mediated by CTLA-4 is the induction of TGF-β production by T cells, which inhibits the production of IFN-γ (89, 90), it has been shown that the stimulation of CD4*^+^* T cells derived from the spleen of animals infected with *L. infantum* results in prominent production of TGF-β. Such a response is not seen when CTLA-4 is blocked, suggesting *in vitro* growth of *L. infantum* depends on the expression of both CTLA-4 and TGF-β, suggesting that the induction of the CTLA-4/TGF-β pathway is important for the replication of the parasite (91). Regarding human disease, the timing of lymphocyte exhaustion is controversial. While Clarencio et al. (92) demonstrated a lower frequency of CTLA-4^+^ T cells in patients with VL, Gautam et al. (43) observed greater expression of CTLA-4 and PD-1 mRNA, important markers of T cell exhaustion, in CD8^+^ T cells. Similar results were observed by Viana et al. (93) and Clarêncio et al. (92). However, CTLA-4 blockade did not alter IFN-γ levels or parasite survival in culture, suggesting that CTLA-4 is not the only molecule responsible for T cell dysfunction during human VL (43). Analysis of the transcriptional profile in CD8*^+^* T cells recovered from peripheral blood from patients also revealed increased expression of CTLA-4 (94). Polymorphisms in the gene encoding CTLA-4 (CTLA-4^+^49-A/G) may be a risk factor for VL development, it was also demonstrated that VL patients who had the polymorphism presented higher anti-Leishmania antibody titers (95). Together, these data indicate that CTLA-4 plays an important role in the T cell exhaustion process observed in VL.

In addition to PD1 and CTLA-4, increased mRNA expression of T cell immunoglobulin and mucin-domain containing-3 (TIM-3) and lymphocyte activation gene-3 (LAG3) is also observed in peripheral blood samples from patients with active VL compared to asymptomatic individuals and endemic controls, which suggests a relationship between the expression of exhaustion markers and disease severity (96). Such receptors are expressed on the lymphocyte membrane after their activation, and when interacting with costimulatory receptors, they interrupt the TCR-dependent cell signaling pathway (97–99), thus maintaining the suppression of the effector response against the parasite and parasite persistence. Studies also demonstrate that LAG-3 and TIM3 are differentially expressed in both natural and induced regulatory T cells (iTregs) and are necessary for their suppressive function (98, 100, 101). In this sense, evaluation of these inhibitory markers on the cell surface is important, as their presence represents the exact moment that T cell exhaustion appears and interferes with the development of protective immunity against the parasite; therefore, they could be biomarkers of disease progression.

## Endogenous Mediators: HIF-1α and Adenosine

The chronic inflammation developed during VL is associated with characteristics that commonly result in a hypoxic microenvironment, such as compromised blood microcirculation and energy demand (102). In this context, there is a reduction in the tissue oxygen supply, leading to the activation of the transcription factor hypoxia inducible factor 1-α (HIF-1α) (103, 104). The role of HIF-1α has been demonstrated during *L. donovani* infection. Parasite infection promotes the increased expression and activation of HIF-1α (105, 106), and macrophages in which HIF-1α is silenced using siRNA control intracellular parasite replication, suggesting that protozoa can use this pathway to survive and proliferate inside the cell (106). The transcription factor IRF-5 compromises the expansion of CD8*^+^* T cells during *L. donovani* infection, and this effect is dependent on HIF1-α in DCs (107). The absence of HIF-1α in DCs results in increased CD8^+^T cell proliferation, enhanced CD4^+^T cell recruitment to the spleen and a pronounced Th1 response, ideal for parasite control, suggesting that HIF-1α may be involved in T cell exhaustion (107, 108). Corroborating these findings, Hammami et al. (109) demonstrated that myeloid cells from *L. donovani*-infected spleens present a phenotype of myeloid-derived suppressor cells (MDSCs) mediated by HIF-1α. Furthermore, HIF-1α appears to be important in the polarization of macrophages into the M2 phenotype, with M2 macrophages being less efficient in controlling the parasite. Conversely, the data suggest that HIF-1α is involved in the persistence of the parasite in the host. Mesquita et al. (110) showed that the absence of HIF-1α increases the susceptibility to infection by *L. donovani*, demonstrating a protective role of HIF-1α during VL. The levels of HIF-1α were higher in both infected C57BL/6 and 129/Sv mice, strains that are naturally resistant to the parasite, than in BALB/c animals, which are susceptible to the disease. Likewise, the absence of HIF1-α results in metabolic dysregulation and increased lipogenesis, which seem to favor parasite growth (110). Although there are contradictory data in the literature, most evidence indicates that HIF1-α favors the persistence of the parasite in the host, acting as a molecule that suppresses protective immunity against pathogens. HIF1-α could also contribute to the persistence of the parasite by acting on the differentiation and functions of Tregs. The role of HIF1-α in regulatory T cells has already been explored by others (111). HIF1-α^-/-^ Tregs lack suppressor function and produce IFN-γ in an excessive manner. The FOXP3 gene promoter in HIF1-α^-/-^ Tregs fails to protect the animal from colitis caused by effector T cells, further demonstrating the role of HIF1-α in the suppressive functions of Tregs (109, 112). To date, the role of HIF1-α in Treg differentiation and functions during VL is not known.

Another endogenous molecule released under conditions of cellular hypoxia is adenosine. Derived from ATP degradation by the action of the ectonucleotidases CD39 and CD73, adenosine is a critical immunosuppressive metabolite released during chronic inflammation and involved in T lymphocyte exhaustion (113–116). In cutaneous leishmaniasis (CL), ADO and AMP act *via* the A2_A_R adenosine receptor to induce tolerogenic dendritic cells (tDCs) through the sequential production of prostaglandin E_2_ (PGE_2_) and IL-10. As a consequence, both mediators inhibit the proliferative ability of CD4^+^ T lymphocytes and IFN-γ production to hinder to the induction of a regulatory profile in such leucocytes, promoting the suppression of the effector immune response against parasites (117). Furthermore, VL patients present elevated serum levels of adenosine, linking ectonucleotidase activity to disease progression (118). Under inflammatory conditions, the A2_B_ receptor is also expressed on monocytes recovered from patients with VL (119), suggesting that during disease, *Leishmania* parasites may use the adenosinergic signaling pathway to avoid the host’s immune response and promote to their own silent growth, thus ensuring their survival inside the cell. In *L. infantum* infection, the parasite benefits from the A2_A_R signaling pathway and promotes the development of an immunosuppressive response mediated by Tregs and IL-10; this inhibits specific Th1 responses, thus allowing the escape of parasites and establishment of infection (58). Increased expression of CD39 and CD73 is observed in effectors and memory T cells with pronounced IFN-γ production and serves to downregulate lymphocyte activation, preventing the host from developing immunopathologies. Soluble factors such as TGF-β and IL-10 can increase the frequency of ectonucleotidases expressed in T cells (117, 120). However, this phenomenon remains to be investigated during VL. The increased and maintained expression of endogenous molecules related to tissue hypoxia, such as HIF-1α and/or the CD39/CD73 ectonucleotidases, may contribute to patient unresponsiveness to conventional drugs applied in the treatment of VL. Such gold-standard drugs not only kill the parasite but also alter host immunity. Antimonial drugs stimulate the production of ROS and NO, while miltefosine and AmBisome induce the secretion of IFN-у, TNF, IL-12, IL-6 and IL1β from leukocytes and reduced anti-inflammatory cytokines (121–124). Thus, both HIF-1α and increased adenosine in the chronic inflammatory microenvironment maintain the constant production of anti-inflammatory cytokines such as IL-10 and TGF-β, promoting the induction of T lymphocyte apoptosis and generation of Tregs; these effects inhibit host immunity and favors the persistence of the parasite, factors that contribute to ineffectiveness of antiparasitic drugs.

## Cytokines

TGF-β is an anti-inflammatory cytokine produced by antigen-specific T cells and by phagocytic mononuclear cells (125). It presents several immunosuppressive effects during infectious diseases, including inhibition of T lymphocyte proliferation, proinflammatory cytokine release and macrophage activation (126). TGF-β inhibits both TNF and IFN-γ function and controls iNOS expression and the development of naïve CD4^+^ T lymphocytes into Th1, Th2, and Th17 cells. TGF-β acts as a facilitator of parasitic growth by modulating both innate and adaptive responses (127) and increasing arginase expression in macrophages (128, 129). In a murine model, TGF-β secreted by lymphocytes in response to *Leishmania* antigens shifts the arginine pool from iNOS to arginase as a source of polyamines, which support parasite growth (130). TGF-β is able to induce apoptosis in lymphocytes isolated from lymph nodes of hamsters infected with *L. donovan*i (131). During *L. infantum* infection, IL-12-deficient C57BL/6 mice show an increased parasite load due to the sustained production of TGF-β in those mice (132).

Another cytokine that plays an important suppressive role in VL is IL-10, and its role in the progression of visceral disease is already well established in both murine and human models. High levels of IL-10 are associated with a higher parasite load and are related to the development of the most severe form of disease (61, 133, 134). Both BALB/c and C57BL/6 mice lacking IL-10 due to either gene deletion or blockade using specific antibodies are resistant to *Leishmania* and do not develop the clinical manifestations of the disease (135–137). In line with these findings, it has been observed that splenic cells aspirated from patients with active VL present higher levels of TNF and IFN-γ under conditions of *in vitro* IL-10 neutralization. Furthermore, such cells present a greater ability to kill parasites (138). Such effects occur because IL-10 is one of the main factors responsible for attenuating the proliferation and activation of T cells, compromising the microbicidal function of macrophages during infection. Impaired immunity is mainly characterized by reduced iNOS expression and NO release (57, 139). Furthermore, the production of IL-10 is associated with T cell differentiation into Th2 cells. Several leucocytes can be sources of IL-10, such as conventional Tregs, Tr1s, CD8^+^ T cells, B cells, NK cells, DCs, macrophages, and neutrophils (60, 61). Among these cells, conventional Tregs, characterized as CD4^+^CD25^+^FOXP3^+^ T cells, and conventional effector T cells, characterized as CD4^+^CD25^-^FOXP3^-^ T cells, are the main sources of IL-10 and are involved in susceptibility to disease. It has been shown that the expansion of IL-10-producing CD4^+^CD25^+^FOXP3^+^ T cells in the plasma of patients with active VL is related to a higher parasite load (64), and there is significant enrichment of this population in bone marrow aspirates from patients with a high parasite load (63). Conditional knockout mice, in which only conventional Tregs do not produce IL-10, present better control of the parasite, although they also display greater disorganization of the splenic microarchitecture (60). Therefore, IL-10 plays a critical role in limiting the antiparasitic immune response by inhibiting the function of important cells involved in parasite restriction.

Several immunoregulatory molecules and pathways, most notably those associated with IL-10 production, are activated following infection by *L. infantum* and *L. donovani* and suppress CD4^+^ T cell functions. One such mechanism includes the induction of IL-10 by BLIMP-1 expressed on Tr1s during clinical and experimental VL. B lymphocyte-induced maturation protein 1 (Blimp-1) is a transcription factor that plays crucial roles in regulating B and T lymphocyte function (140, 141). In different models of inflammatory disease (i.e., asthma, colitis, and infection), mice with specific Blimp-1 gene deletion in T lymphocytes show pronounced production of cytokines, which contributes to worsening inflammation (142–145). IL-10 produced by Tr1s through the BLIMP-1 pathway suppresses specific immunity to the parasite but plays a critical role in protecting the tissue against inflammatory insults induced during the chronic process caused by the parasite (146).

Type I IFNs, including IFN-α and IFN-β, bind to the IFN_A_R receptor and display regulatory functions preventing pathogen control. IFNα/β remains elevated during chronic infectious processes and induces the expression of IL-10, indoleamine 2,3-dioxygenase 1 (IDO-1), PDL1 and other negative regulators of T cell responses, such as TIM3, in CD4^+^ T lymphocytes, in addition to their role in promoting apoptosis of T cells *via* Fas/FasL (147, 148). In experimental infection caused by *L. infantum*, type I IFNs (IFN-α and IFN-β) produced during parasite recognition *via* sequential signaling mediated by TLR4/TRIF/IRF-1 suppress the development of Th1 responses *via* mechanisms depending on IL-10, which contributes to antigen persistence. Patients with classical symptoms of VL present reduced expression of genes associated with TLR4 and IFN-I compared to asymptomatic individuals and endemic controls, suggesting that failure of the regulatory mechanisms of the immune response favors the exacerbation of inflammation and, consequently, leads to the most severe form of disease (31). Likewise, during *L. donovani* infection, IFN-I contributes to parasite persistence in the target organs of visceral disease by suppressing the development of the Th1 response and promoting Tr1 expansion. Interestingly, temporary blockade of the type I IFN-mediated signaling pathway ameliorated the therapeutic efficacy of antiparasitic drugs by increasing tolerance of the parasite (149). In both mentioned studies, the improvement of parasite replication control in the absence of the type-I IFN signaling pathway was dependent on a pronounced Th1 response generated in the infected tissues. However, this control was accompanied by an enormous cost to the host, since the aberrant inflammation generated promoted liver damage while the infection progressed (31, 149).

IFN-γ also displays an immunosuppressive effect. Persistent presence of the parasite maintains high levels of IFN-γ, contributing to cell exhaustion by inducing IDO. This enzyme catalyzes the breakdown of tryptophan into kynurenine, inducing apoptosis of T lymphocytes by activating the caspase-8 pathway and releasing mitochondrial cytochrome c (150). Conventional Tregs expressing IDO, PDL1 and CTLA4 are present in the peripheral blood of cancer patients and are strongly related to the severe forms of the disease (151, 152). Interestingly, IDO enzymatic activity in human VL is associated with immunosuppression and can be used as a biomarker of human disease caused by *L. infantum* (153). In the model of CL caused by *L. major*, IDO is a key factor attenuating the inflammatory response and increasing parasite replication. Administering IDO inhibitors to mice with well-established infection reduces the parasite load associated with inflammation (154), suggesting that IDO inhibitors may offer a new therapeutic tool for patients with chronic leishmaniasis. IDO inhibitors are already being used in clinical oncology as adjuncts to broaden the therapeutic window and limit the autoimmune side effects that immunobiological therapy can cause in cancer patients (155, 156).

Other cytokines have been implicated in inducing the expression of inhibitory receptors on leukocytes. In this context, the role of IL-27 in increasing TIM3 expression in CD4^+^ T cells has been illustrated (148). *In vivo*, the maintained expression of IL-27 promotes the enhancement of inhibitory receptors on T cells, such as PDL1, LAG3, TIGIT and TIM3 (97, 157, 158). IL-27 is composed of the EBI-3 and p28 subunits, belongs to the IL-6/12 cytokine family and was originally described as a cofactor for Th1 lymphocyte differentiation, together with IL-12 (159, 160). The mechanism by which IL-27 induces inhibitory receptors remains to be determined, but it is proposed that stimulation of TCRs by persistent antigens increases chromatin accessibility, allowing IL-27-induced STAT1 to bind directly upstream of the PD-L1, LAG-3, CTLA-4, TIGIT and TIM3 gene promoters (161). Furthermore, IL-27-mediated signaling through IL-27Rα (an IL-27 receptor) is responsible for the upregulation of TIGIT and PD1 in memory T cells during infectious processes, such as toxoplasmosis and malaria, and cancer (97, 158). In a sepsis model, IL-27Rα expression was associated with TIGIT but not with PD1 expression in memory CD4^+^ T cells and the loss of IFN-γ production (162). During VL caused by *L. infantum*, the absence of IL-27 promotes prominent Th1 inflammation that causes immunopathology (163). Quirino et al. (163) demonstrated that IL-27 is able to induce IL-10-producing Tr1s and plays an important regulatory role in mediating susceptibility to infection. IL-27 inhibits the production of IL-17A in infected tissue and controls neutrophil influx to target organs, preventing the development of liver injury but favoring parasite growth (163). Similarly, IL-27R-deficient mice infected with *L. donovani* develop immunopathology due to an increased Th1 response in VL organs (164). Patients with active VL show an increase in systemic IL-27 (163, 165), and IL-27 in the presence of IL-21 promotes the expansion of specific IL-10-producing T cells (45, 166). However, it remains unclear whether IL-27 suppresses the specific immune response of T cells in VL by promoting the expression of multiple inhibitory receptors on CD4^+^ T and CD8^+^ T lymphocytes.

IL-27, composed of p28 and EBI3, shares common subunits with IL-35 (p35 and EBI3) and IL-39 (p19 and EBI3) (167, 168). IL-35 has been reported to have immunosuppressive activity in autoimmune diseases, cancer and infectious diseases (169–172). In VL caused by *L. donovani*, the chronic inflammatory response is accompanied by enhanced Tregs, TGF-β and IL-35, factors that suppress all T cell effector functions. TGF-β and EBI-3 act synergistically to inhibit the Th1 response and maintain parasite persistence. Double neutralization using specific antibodies against EBI3 and TGF-β limits progression of the infection by promoting remarkable effector immunity against the parasite (172). In the chronic phase of VL caused by *L. donovani*, Tregs suppress the proliferation and functions of Th17 cells *via* mechanisms that depend on TGF-β and IL-35, preventing disease progression but maintaining parasite persistence. Restoration of the IL-17 response during *in vivo* neutralization of TGF-β and IL-35 is accompanied by hepatic and splenic resistance consistent with the low parasite load in such organs (173). IL-35 production by Tregs has been shown to play a central role in T cell exhaustion, as indicated by its ability to induce the expression of LAG3, TIM3, and PD1 on the cell surface of tumor-infiltrating CD4^+^ T and CD8^+^ T cells (174), and it is a topic that warrants exploration in experimental and human VL.

## Conclusion and Future Perspectives

**Figure 2:**
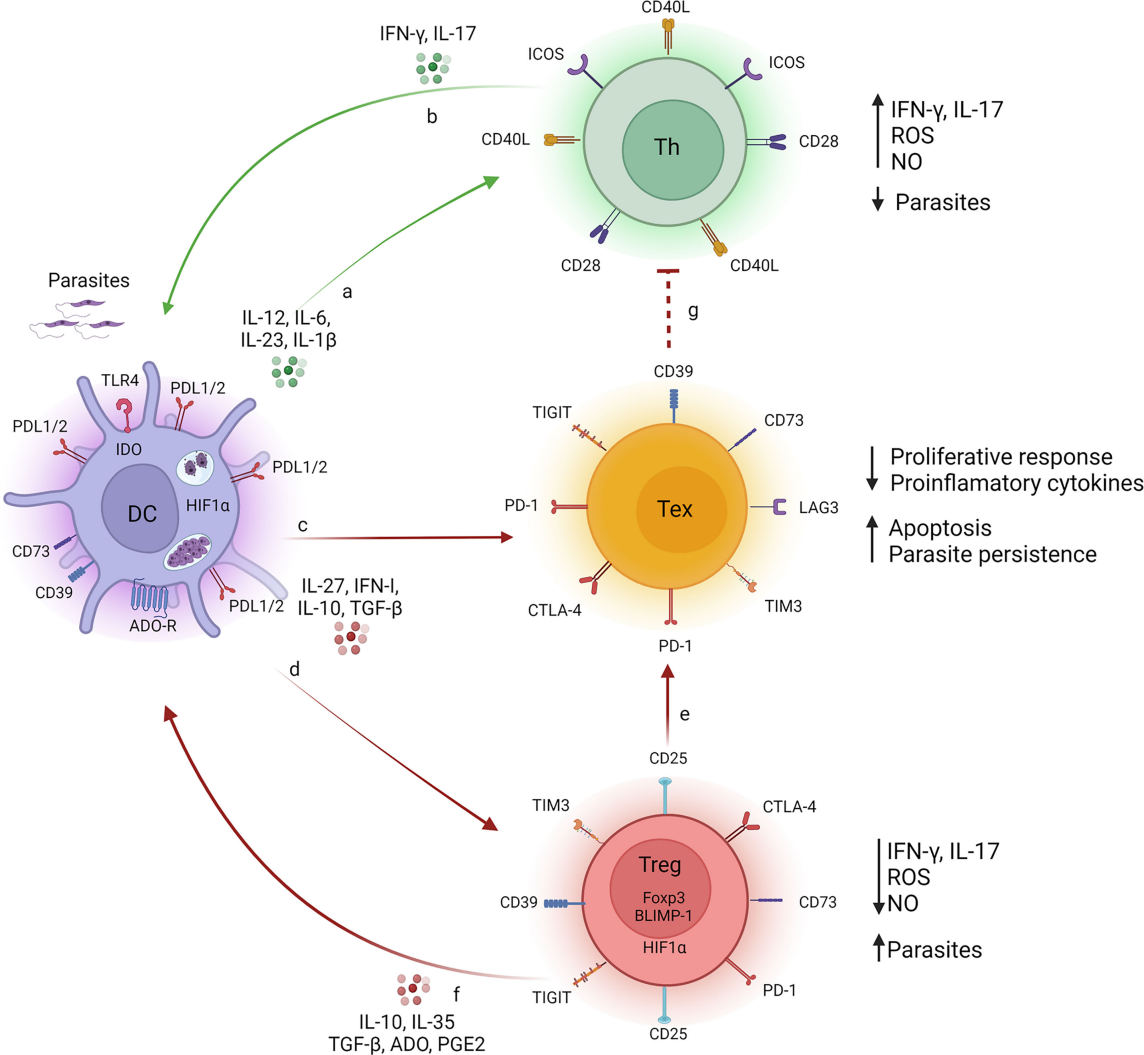
T cell fate during Visceral Leishmaniasis. During infection with *L. donovani* or *L. infantum*, monocyte-derived cell (i.e. dendritic cell or macrophage) (purple cells) produce pro-inflammatory cytokines (IL-1β, IL-6, IL-12 and IL-23), which act on naive T lymphocytes, targeting the cells to Th1 and Th17 subtypes (green cells), resulting in the production of IFN-γ/IL-17 **(A)**. Secreted IFN-γ and IL-17 promote the phagocytes microbicidal function of monocyte-derived cell trough the release of ROS and NO, reducing the parasite load **(B)**. The persistent presence of parasites induces a sustained and maintained production of regulatory molecules, such as TGF-β, IL-10, IL-27, PGE2, which promote exhaustion of T lymphocytes (yellow cells), a process characterized by increased expression of inhibitory molecules, such as CTLA-4, PD-1, TIM3, TIGIT, CD39, CD73, LAG-3, on T cell surface. As a consequence, the proliferative response and production of pro-inflammatory cytokines are suboptimal, mechanisms by which the parasite uses to survive in the host-vertebrate **(C)**. Such regulatory molecules influence the inhibitory receptors on the surface of Treg cells (red cells) impacting on their both differentiation and suppressive function **(D)** and upon DCs functions **(F)**. Such factors repress the protective immune response mediated by IFN-γ/IL-17 and ROS/NO **(G)**. Tregs also respond to regulatory molecules, and contribute to the exhaustion process of T lymphocytes by sustaining the release of regulatory mediators (TGF-β, IL-10, IL-35, PGE2 and adenosine) in the microenvironment and maintaining high levels of inhibitory molecules on the surface of exhausted T lymphocytes **(E)**. Together, these signs favor the persistence of the parasite. Legends: green arrow (induction of effector response), red arrow (induction of suppressor response), red dotted arrow (inhibition of effector response). Created with BioRender.com (Agreement number: LC23QKDV88).

VL is a serious public health problem, causing high morbidity and mortality in several countries. Among infected individuals, 85% remain asymptomatic, while the other 15% present clinical manifestations, ranging from oligosymptomatic (mild) forms to more severe symptomatic forms that can lead to death if not properly treated. In the absence of effective vaccine strategies, therapy with pentavalent antimonial and amphotericin B is routinely used for VL treatment. However, these drugs present adverse reactions and side effects and require several rounds of treatment, promoting the acquisition of resistance with long-term use (8, 175, 176). An important question that arises is whether the refractoriness of critically ill patients is a consequence of combined and redundant actions of factors that promote the exhaustion of T lymphocytes. The absence of the proliferative response of T lymphocytes to parasite antigens and the inability to produce IFN-γ indicate that cell exhaustion is occurring, and a more accurate assessment should be performed when these features manifest (177). As such, combining immunotherapy, specifically drugs targeting inhibitory molecules and regulatory cytokines, with drugs routinely used earlier in the process when T cell exhaustion appears could reduce the chances of VL patients acquiring resistance to conventional treatments. Such strategies have been gaining attention from the scientific and medical communities (124, 178–180). The optimization of treatments combining typical drugs with immunotherapy might help to delay the emergence of resistance and increase the therapeutic lifespan of the respective drugs. Immunotherapy focused on inhibitory molecules of the immune system, also known as modulators of immune checkpoints, has revolutionized the oncology field, and responses far beyond the remarkable clinical effectiveness in some patients have been achieved. The prevalence of immunotherapy has generated a dramatic change in how the efficacy and toxicity of antineoplastic treatment are evaluated, with a more holistic view of cancer patients. An example of a drug used successfully in clinical oncology is ipilimumab, the first immunological checkpoint-blocking antibody (blocking CTLA4) to be authorized for clinical use. The approval of ipilimumab was quickly followed by the development of monoclonal antibodies targeting PD1 (pembrolizumab and nivolumab) and PDL1 (atezolizumab and durvalumab). Currently, anti-PD1/PDL1 antibodies have become one of the most widely prescribed anticancer therapies (181). The first studies of immunotherapies as treatments for experimental VL came from Murray et al. (57), who reported that anti-IL-10 receptor monoclonal antibodies (mAbs) promote parasite death *via* iNOS-dependent mechanisms and increase IFN-γ expression. The results obtained from studies of experimental disease models and in human samples suggest that immunotherapy involving different antagonists of the inhibitory molecules involved in the course of disease progression is a promising treatment option for VL. As explored throughout this review, blocking the inhibitory targets of the immune system rescues the host from the subversion of the immune response imposed by the parasite to successfully establish the infection in the organism, leading to healing (**Figure 2**). Thus, combining inhibitory molecules that block the immune system with conventional drugs used for the treatment of VL could lead to promising results and increase the identification of therapeutic targets that restore the host’s initial defense mechanisms, preventing disease progression. We recognize that the clinical potential in this field is broad and that enormous research efforts and rapid refinement of the understanding of the role of immune system inhibitory molecules in experimental and human diseases are needed.

## Author Contributions

Conceptualization: VC; Writing - Original Draft: JC-M, GT, PA, and VC; Writing - Review and Editing: JS and VC; Funding Acquisition: JS and VC; Resources: JS and VC; and Supervision: VC. All authors contributed to the article and approved the submitted version.

## Funding

We are thankful to FAPESP, CNPq for their financial support. The research leading to these results has received funding from the São Paulo Research Foundation (FAPESP) under grant agreements N 2019/12991-8 and N.° 2013/08216-2 (Center for Research in Inflammatory Disease) and Universal Project (CNPq) under agreement N° 302419/2018-7 from the University of São Paulo.

## Conflict of Interest

The authors declare that the research was conducted in the absence of any commercial or financial relationships that could be construed as a potential conflict of interest.

## Publisher’s Note

All claims expressed in this article are solely those of the authors and do not necessarily represent those of their affiliated organizations, or those of the publisher, the editors and the reviewers. Any product that may be evaluated in this article, or claim that may be made by its manufacturer, is not guaranteed or endorsed by the publisher.
